# Three Cases of Right Heart Thrombus: Using POCUS for the Diagnosis of Thromboembolism in COVID-19

**DOI:** 10.24908/pocus.v7i2.15534

**Published:** 2022-11-21

**Authors:** Sergio Velasco Malagón, Juliana Moreno Ladino, Hector Andres Ruiz

**Affiliations:** 1 Internal Medicine Department, Universidad Nacional de Colombia Colombia; 2 Grupo de Interés en Ultrasonografía Enfocada UN-HUN; 3 Geriatric’s Unit, Universidad Nacional de Colombia Colombia; 4 Critical Care Department, Hospital Universitario Nacional de Colombia Colombia

**Keywords:** SARS-CoV-2, POCUS, Pulmonary embolism

## Abstract

COVID-19 generates a series of challenges, one of them being thrombotic manifestations of the disease. The growing use of POCUS and its wide versatility have expanded its use outside of radiology rooms. The development of focused protocols has facilitated its use in emergency units, clinical wards, intensive care units, and operating rooms. We report three cases of patients with SARS-CoV-2 infection in whom the use of POCUS allowed the identification of the presence of intracavitary thrombus with acute right ventricular dysfunction. These cases illustrate the importance of ultrasound focused on critically ill patients in guiding the diagnosis and treatment amid the pandemic situation.

## Background

Infection with SARS-CoV-2 increases the risk of venous thromboembolism 1. The development of POCUS protocols has facilitated its use in emergency units, clinical wards, intensive care units, and operating rooms. Herein are three cases of patients with SARS-CoV-2 infection in whom the use of POCUS facilitated the diagnosis of right heart thrombus and acute right ventricular dysfunction. These cases illustrate the importance of POCUS in critically ill patients.

## Case 1

**Figure 1:**
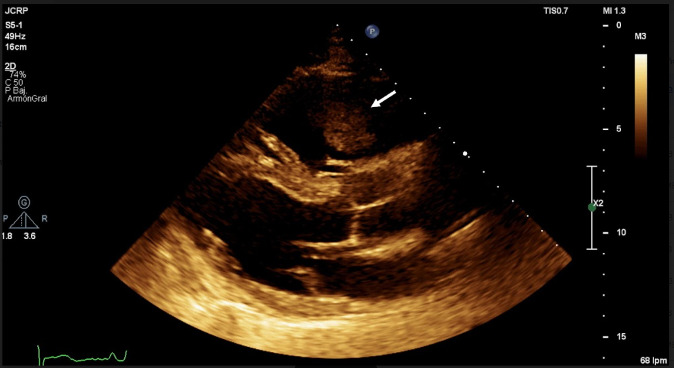
PLAX showing a clot in transit in the right ventricle (arrow).

A 46-year-old male patient with a history of type 2 diabetes mellitus and morbid obesity (BMI 51.9) presented with a chief complaint of paroxysmal dry cough and occasional hemoptysis for the past week, he denied fever or shortness of breath, and the diagnosis of COVID-19 was made using a specific PCR test. Upon admission, he had a PaO2 / FiO2 ratio of 64, heart rate of 70, respiratory rate of 15, blood pressure of 129/75 mmHg. Cardiopulmonary auscultation did not reveal significant findings or signs of respiratory distress. POCUS exam was then performed: the vena cava was not fully visualized due to gas interposition in the subxiphoid window. In the long parasternal axis, there was abnormal movement of the interventricular septum, decreased left ventricular function, and septal separation of Point E (EPSS <10), surprisingly a right heart thrombus in the right ventricle was also seen. The short parasternal axis window showed a dilated right ventricle and flattening of the interventricular septum as well as a hyperechoic image in the pulmonary artery (Figure 1 and Online Video S1). Pulmonary ultrasound showed confluent B-lines predominantly in the right anterior quadrants; the posterior quadrants revealed a basal consolidation and superior confluent B-lines. Pleural effusion was not observed. Computed tomography pulmonary angiography (CTPA) confirmed acute thromboembolism in the right main branch of the pulmonary artery, chronic segmental and subsegmental thromboembolism, as well as images suggestive of viral pneumonia. The transthoracic echocardiogram performed by the cardiology service of the hospital showed a good correlation with the first POCUS examination. Anticoagulation with low molecular weight heparin was started, and since the patient did not deteriorate during follow-up monitoring, he was transferred to the hospital floor.

## Case 2

**Figure 2:**
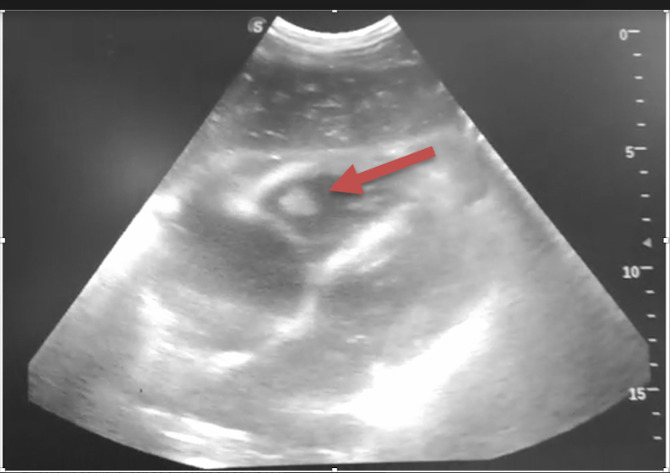
Subxiphoid view showing a clot in transit in the right ventricle (arrow).

A 64-year-old male patient with no significant medical history presented to the emergency service with four days of fever, dry cough, and progressive shortness of breath. He was admitted to the emergency room with a heart rate of 122, respiratory rate of 34, blood pressure of 101/62 mmHg, oxygen saturation of 82% on room air. On auscultation, rales were detected in both lung fields; his heart sound showed tachycardia with no murmurs or rubs. The patient was somnolent, inattentive, disoriented, and his capillary refill time was around 3 seconds. In the resuscitation area, mechanical ventilation was initiated with a suspected diagnosis of primary acute respiratory distress syndrome (ARDS) due to viral pneumonia, using an intubation sequence with fentanyl and midazolam. The patient presented cardiac arrest during induction with a pulseless electrical activity rhythm, requiring standard resuscitation with institutional protected Advanced Cardiac Life Support (ACLS) protocol with return of spontaneous circulation (ROSC) after 2 minutes. An adverse effect of fentanyl was suspected. He required high doses of norepinephrine to maintain mean arterial pressure of 65 mmHg. He was admitted to the intensive care unit, where POCUS evaluation was performed, finding B-lines in both lung fields, ventricular interdependence, peak systolic velocity of the right ventricular outflow tract of 74.5 cm/s, PVAT (pulmonary acceleration time) 90 ms, TAPSE (systolic excursion of the tricuspid annulus) of 14 mm, the diameter of the inferior vena cava greater than 2 cm without inspiratory collapse and in the subxiphoid cardiac window, an image adjacent to the tricuspid valve apparatus compatible with a right heart thrombus (Figure 2 and Online Video S2). Given the instability and suspicion of SARS-COV-2 infection, he was not transferred to CT angiography, and a presumptive diagnosis of acute pulmonary embolism with ventricular dysfunction was made. Fibrinolysis with alteplase was performed, with subsequent improvement in ventricular interdependence, increased TAPSE value, disappearance of the image described in the right ventricle, and tolerance to vasopressor withdrawal. Subsequently, there was a positive RT-PCR test for SARS-COV-2 infection, and the diagnosis of pulmonary embolism was confirmed by CT angiography.

## Case 3

**Figure 3:**
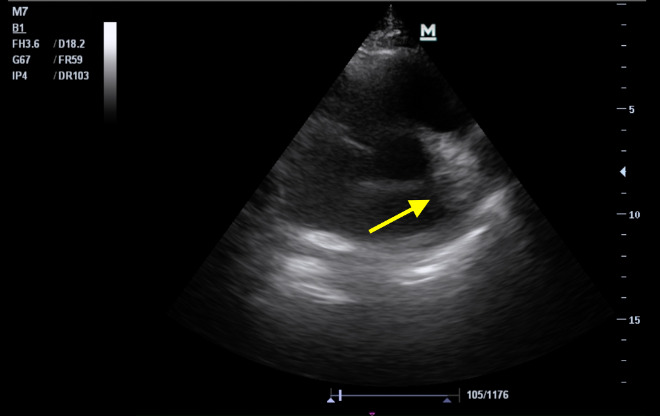
PSAX view showing a clot in the pulmonary artery.

A 35-year-old male patient with no significant medical history was admitted to the hospital with severe SARS-COV-2 pneumonia; prognostic blood markers showed elevated LDH and CRP; troponin and D-dimer were negative. High-flow nasal cannula therapy was used initially. However, the patient persisted with severe hypoxemia, and pulmonary embolism was then considered as a differential diagnosis. Formal Doppler ultrasound for the lower extremities was performed, ruling out deep venous thrombosis. Given high clinical suspicion and no other clinical cause for his decline, anticoagulation with low molecular weight heparin was started. Later the patient progressed to respiratory failure with sudden hemodynamic deterioration, hypotension, altered state of consciousness, and eventually cardiac arrest requiring standard ACLS resuscitation with ROSC at 4 minutes. He was then admitted to the ICU requiring ventilatory support and dual vasopressor with norepinephrine and vasopressin. Upon admission, POCUS was performed with evidence of severe dilation of the right ventricle with ventricular interdependence and qualitative dysfunction; in the short parasternal axis, at the pulmonary valve level, there was a hyperechoic image in the main trunk of the pulmonary artery (Figure 3 and Online Video S3). Since the patient was unstable, CT scan was deferred, but the diagnosis of massive pulmonary embolism was considered very likely, and fibrinolysis was performed using 50 mg of tenecteplase. Additional protective ventilatory parameters were set for the right ventricle. Chest CT angiography was performed and the diagnosis was confirmed, but unfortunately, the patient presented a new event of cardiac arrest and, due to the unavailability of ventricular support strategies, the patient died.

## Discussion points

Less invasive imaging, with no radiation and easily portable tools, are found to be extremely useful in the context of the COVID-19 pandemic. POCUS has been used for the diagnosis of pulmonary embolism, achieving a remarkable diagnostic performance, ideal for these patients in which a mechanism of shock cannot be easily elucidated and in whom additional workup is limited given the patient clinical instability. In addition, POCUS, as shown in this case series, can be used to assess patients with undifferentiated shock and dyspnea 2. Its use has also been referenced in several publications to guide the diagnosis and management of patients with pulmonary embolism 3, 4. Furthermore, POCUS decreases time to intervention and improves outcomes in this setting of patients 5.

Thrombotic complications are often associated with worse clinical outcomes. For instance, the incidence of venous thromboembolism is 3-4 times more common in patients with COVID-19 vs. non-COVID-19 population, and the prevalence is nearly up to 30%, despite having received continuous thromboprophylaxis 6.

A right heart thrombus is found in less than 4% of patients with pulmonary embolism and carries a worse clinical prognosis, with mortality reported in 44.7% 7. The most frequent ultrasound signs found in pulmonary embolism are dilation of the right ventricle (27.4%), right ventricular free wall hypokinesia (26.6%), and ventricular interdependence (18.4%) 8. To further evaluate the right ventricular function, some advanced measurements can also be performed by trained physicians, such as pulmonary acceleration time and systolic excursion velocity of the right ventricle (RV S’). The cutoff value of RV S' is <11.5 cm/s and highly correlates with poor RV function with a sensitivity of 90% and specificity of 85%  9. According to the European Society of Cardiology (ESC)^10^ recommendations, it is reasonable to perform a POCUS-guided intervention when diagnosis with gold-standard CT angiography can't be done. As evidenced in the above-mentioned cases, this strategy drastically helped the clinical team make quick clinical interventions.

In the cases presented in this article, POCUS displayed an acute right ventricular dysfunction in the setting of right heart thrombus suggesting pulmonary embolism as the cause of hemodynamic dysfunction, allowing early treatment in the ICU. Though the patient in case 1 was not in shock, early diagnosis led to prompt anticoagulation and minimized further derangements. In case 2, POCUS supported the diagnosis of pulmonary embolism in the settlement of cardiac arrest against the previous suspicion of fentanyl adverse effect. It also granted the unique opportunity of follow-up showing the resolution of intraventricular interdependence and the disappearance of the thrombus inside the right ventricle.

In case 3, POCUS showed a thrombus in the main pulmonary artery and confirmed diagnosis of pulmonary embolism as the cause of shock, and lysis was used in an attempt to unload the right ventricle. However, the severity of the clinical scenario and the insufficient means to deliver ventricular assistance were imminent and lastly, the patient died.

As mentioned above, the gold standard of pulmonary embolism implies the use of CT scan. Still, during COVID-19 this approach was limited for two main reasons: the often-clinical instability of the patients with confirmed SARS-COV-2 infection and the high risk of infection among health care providers. For that reason, POCUS emerges as an appealing alternative to diagnose and guide management in these patients who need fast and reliable interventions in a particular setting where there is a special need to minimize unnecessary viral exposure. In summary, these cases highlight the importance of POCUS in critical care settings during COVID-19 pandemic.

## Statement of ethics

The authors certify that verbal patient consent has been obtained for the images. The clinical information did not use personal information and was reported to be published in a medical journal to enhance cooperative academic discussions in this unexpected worldwide crisis. The patients understand that their name, initials, or any identifying information will not be disclosed and won't be published.

## Disclosure

The authors declare no conflicts of interest.

## Supplementary Material

 Video S1 VPLAX showing a clot in transit in the right ventricle (arrow).

Video S2 Subxiphoid view showing a clot in transit in the right ventricle (arrow).

Video S3 PSAX view showing a clot in the pulmonary artery.

## References

[R165879526892897] Malas M B, Naazie I N, Elsayed N, Mathlouthi A, Marmor R, Clary B (2020). Thromboembolism risk of COVID-19 is high and associated with a higher risk of mortality: A systematic review and meta-analysis. EClinicalMedicine.

[R165879526892898] Bagheri-Hariri S, Yekesadat M, Farahmand S, Arbab M, Sedaghat M, Shahlafar N, Takzare A, Seyedhossieni-Davarani S, Nejati A (2015). The impact of using RUSH protocol for diagnosing the type of unknown shock in the emergency department. Emerg. Radiol.

[R165879526892895] Goldhaber S Z (2002). Echocardiography in the Management of Pulmonary Embolism. Ann. Intern. Med.

[R165879526892891] Vieillard-Baron A, Page B, Augarde R, Prin S, Qanadli S, Beauchet A, Dubourg O, Jardin F (2001). Acute cor pulmonale in massive pulmonary embolism: incidence, echocardiographic pattern, clinical implications and recovery rate. Intensive Care Med.

[R165879526892894] Zieleskiewicz L, Lopez A, Hraiech S, Baumstarck K, Pastene B, Bisceglie M Di, Coiffard B, Duclos G, Boussuges A, Bobbia X, Einav S, Papazian L, Leone M (2021). Bedside POCUS during ward emergencies is associated with improved diagnosis and outcome: an observational, prospective, controlled study. Crit. Care.

[R165879526892896] Klok F A, Kruip Mjha, Meer Njm Van Der, Arbous M S, Gommers Dampj, Kant K M, Kaptein Fhj, Paassen J Van, Stals Mam, Huisman M V, Endeman H (2020). Incidence of thrombotic complications in critically ill ICU patients with COVID-19. Thromb. Res.

[R165879526892889] Kahl N, Gabriel C, Lahham S, Thompson M, Hoonpongsimanont W (2019). Point-of-care Ultrasound Diagnosis of Pulmonary Embolism with Thrombus in Transit. Clin. Pract. Cases Emerg. Med.

[R165879526892893] Kurnicka K, Lichodziejewska B, Goliszek S, Dzikowska-Diduch O, Zdończyk O, Kozłowska M, Kostrubiec M, Ciurzyński M, Palczewski P, Grudzka K, Krupa M, Koć M, Pruszczyk P (2016). Echocardiographic Pattern of Acute Pulmonary Embolism: Analysis of 511 Consecutive Patients. J. Am. Soc. Echocardiogr.

[R165879526892890] Rudski L G, Lai W W, Afilalo J, Hua L, Handschumacher M D, Chandrasekaran K, Solomon S D, Louie E K, Schiller N B (2010). Guidelines for the Echocardiographic Assessment of the Right Heart in Adults: A Report from the American Society of Echocardiography. J. Am. Soc. Echocardiogr.

[R165879526892892] Konstantinides S V, The Meyer G (2019). ESC Guidelines on the Diagnosis and Management of Acute Pulmonary Embolism. Eur. Heart J.

